# Aerosol Transmission of Norovirus

**DOI:** 10.3390/v16010151

**Published:** 2024-01-19

**Authors:** Mengdi Tan, Yi Tian, Daitao Zhang, Quanyi Wang, Zhiyong Gao

**Affiliations:** 1 School of Public Health, China Medical University, Shenyang 110122, China; 2 Institute for the Control of Infectious and Endemic Diseases, Beijing Center for Disease Prevention and Control, Beijing 100013, China

**Keywords:** acute gastroenteritis, norovirus, aerosol, transmission

## Abstract

Norovirus (NoV) is a major cause of acute gastroenteritis outbreaks worldwide. A comprehensive understanding of the transmission mode is of great significance for the prevention and control of the NoV infection. Currently, the transmission modes of NoV include contact, food-borne, water-borne and aerosol transmission. The first three modes are more common, while aerosol transmission is seldom reported. In this paper, the source, generation mechanism, infectivity, sampling and related outbreaks of NoV aerosol are summarized and discussed.

## 1. Introduction

Norovirus (NoV) is an RNA virus belonging to the *Caliciviridae* family. Human NoV is currently considered to be the major cause of acute gastroenteritis (AGE) in all age groups worldwide, estimated to account for one-fifth of all gastroenteritis cases globally and approximately 699 million infections per annum [1,2]. NoV is transmitted through the fecal–oral route, including direct person-to-person contact, contact with contaminated environmental surfaces, ingesting contaminated water or food, and aerosol transmission [3,4,5]. Aerosol transmission is considered a less common mode of transmission compared to contact, foodborne and waterborne transmission, and there are relatively few reports on NoV aerosol transmission, as well as a lack of summary analysis. The most common symptoms of the NoV infection are diarrhea and vomiting. The infectious dose of NoV is very low, as little as 18 viral particles can cause infection, and the presence of a large number of viral particles in the feces and vomit of infected persons makes NoV highly contagious [6,7,8]. NoV tends to cause gastroenteritis outbreaks in semi-enclosed and closed environments such as nursing homes, schools, hospitals, restaurants, cruise ships, child-care centers, militaries, etc. [9,10]. Recognizing the pivotal role of aerosol transmission in NoV outbreaks is crucial for formulating effective prevention and control strategies. This article reviews the sources, generation mechanism, infectivity, sampling and related outbreaks of NoV aerosol.

## 2. Production and Characteristics of Bioaerosol

Aerosol refers to a gaseous dispersion system composed of solid or liquid particles suspended in a gaseous medium (such as air) [11]. Bioaerosol is when viruses, bacteria, fungi and other microorganisms attach to aerosol particles [12].

The movement of aerosols in the environment is affected by gravity and airflow. The settling velocity of aerosols is proportional to the square of the particle diameter in still air, making larger particles settle more rapidly than the smaller ones [11]. The size of aerosol particles not only determines their residence time in the air but also the probability of being inhaled by humans [13]. The particle sizes of bioaerosols vary greatly, and disease-related microbial aerosols in the environment are mainly concentrated in 0.1~20 μm in diameter [14]. At present, WHO takes 5 μm as the dividing line, and the particles with a diameter greater than 5 μm are classified as droplets and ≤5 μm as droplet nuclei [15]. Notably, previous studies have posited that aerosol particles within the 5 to 10 μm range could remain suspended in the air for dozens of minutes, spread a certain distance in the airflow, and were easy to be inhaled to reach the lower respiratory tract of a human. The aerosol particles of ≤5 μm had a longer suspension time in the air, a longer transmission distance and can reach the lungs or even alveoli through the respiratory tract [16,17].

Bioaerosol transmission is commonly recognized as a transmission mode for respiratory viruses, with limited comprehensive investigations focusing on gastrointestinal viruses. Due to the challenges associated with NoV culture and the limited efficiency of bioaerosol samplers in collecting these viruses, the transmission mechanism of NoV aerosols remains unclear.

## 3. Sources of NoV Aerosols

NoV aerosol particles are mainly derived from vomiting and diarrhea of AGE patients infected with NoVs.

### 3.1. NoV Aerosol from Vomiting of Infected People

From the perspective of biological rationality, the occurrence of NoV aerosol transmission should meet three conditions [12]. First, the vomiting of infected people can produce bioaerosols containing NoVs. Vomiting is one of the main symptoms of the NoV infection, and there are a large number of viral particles in the vomit of infected people [18,19,20]. Aerosols are formed in two main ways throughout vomiting. One is that the pressure of the vomiting action itself can atomize the liquid containing the viral particles [21]. The other is that the viral particles are caught by mechanical movement or airflow. The vomit contaminates the surface of the ground or other objects, and the airflow around the contaminated objects disperses the virus into the air [22]. Ciofi-Silva et al. [23] found that NoVs could also be aerosolized from contaminated floors during mopping, with an average viral concentration of 205 genome copy numbers/m^3^. Previous studies had found that many NoV outbreaks occurred in hotels, schools and cruise ships, and that they were closely related to the exposure to the vomit of infected persons. Moreover, NoV may be transmitted through the inhalation of aerosol produced by vomiting [24,25,26,27]. The results of Marks et al. [24] showed that, in a hotel restaurant, the infection risk of diners was highly correlated with the distance between their seats and the location of vomiting, i.e., the greater the distance, the lower the risk of infection, and other transmission modes were ruled out in epidemiological investigations. In addition, in the study of Tung-Thompson et al. [28], a device scaled to human physiological parameters was used to simulate the process of vomiting, and it was proved that phage (MS2), a substitute for human NoV, did indeed undergo aerosolization during vomiting. Subsequently, Alsved et al. [13] also collected NoV-positive air samples from hospitals where NoV outbreaks happened. In their study, the concentration of NoV in the air ranged from 5 to 215 genomic copy numbers/m^3^, and the concentration of NoV RNA in the air was strongly correlated with the time of the last vomiting episode, suggesting that recent vomiting was the main source of NoVs in the air. This study also suggested that airborne particles smaller than 1 μm contained detectable NoVs, which could be easily inhaled by individuals and had a 10% to 30% probability of deposition in the mouth, nose or trachea. In this scenario, the virus was likely to be further swallowed and transported to the gastrointestinal tract.

Second, NoV can exist in the external environment for a certain amount of time. Kramer et al. [29] demonstrated that gastrointestinal viruses such as NoV, astrovirus and rotavirus can persist in the environment for several weeks without regular preventive disinfection. Bonifait et al. [30] confirmed the presence of NoV in the air in medical institutions during outbreaks, indicating that NoV can be suspended in the air for a long period, with NoV concentrations ranging from 1.35 × 10^1^~2.35 × 10^3^ genome copy numbers/m^3^ and above. In addition, NoV aerosols have been detected in wastewater treatment plants, medical facilities and biosolid land application sites, suggesting that NoV aerosols can be present for some time in a variety of settings [30,31,32,33].

Third, NoV aerosols are contagious. Bonifait et al. [30] used the murine norovirus (MNV) and NIOSH-251 cyclone air sampler to evaluate the infectivity of NoV aerosols in vitro and found that the aerosolized MNV-1 preserved its infectivity and integrity. However, Alsved et al. [34] used two aerosol generators to produce MNV aerosols and collected aerosols with a liquid impact sampler and found that the infectivity of both aerosols decreased. The different results may be related to different air samplers, and different sampling mechanisms may affect the active state of viruses in aerosols. At present, there is still a lack of mature in vitro culture systems for NoV [33], and there is no gold standard method or instrument for sampling NoV aerosol [35]. Therefore, the infectivity research of NoV aerosols has been hampered. To address the challenges in evaluating NoV infectivity, researchers have proposed more accessible alternative techniques, such as molecular assays, animal models and surrogate virus systems, to gain insights into the viability and infectivity of the virus [36,37,38,39].

### 3.2. NoV Aerosol from Diarrhea of Infected People

If the diarrhea of an infected person is capable of causing NoV aerosol transmission, three conditions also need to be met. First, NoV aerosol can be produced when an infected person has diarrhea. The latter two were described above. Diarrhea is one of the main symptoms of the NoV infection, and the feces of infected persons also contain a large amount of viral particles [6]. The aerosols have two sources during the course of diarrhea. First, NoV-infected persons usually present with an acute, watery and recurring diarrhea symptom; thus, NoV aerosol is likely to be produced during stressful defecation. However, there are few studies on aerosol generation during diarrhea, and the concentration of NoV aerosol and its correlation with the severity of diarrhea remains unknown. The second is that NoV aerosols can be produced when infected individuals flush the toilet after defecation [40]. The results of Johnson et al. [41] showed that aerosols can be generated in the flushing process of the toilet bowl, and aerosolization can still occur even after multiple flushes. Similarly, two studies by Barker and Knowlton et al. [42,43] also found that the bacteria and viruses in toilet water can be aerosolized during flushing. In addition, Schreck and Verani et al. [44,45] found that toilets were the main source of viral pollution: when contaminated toilets are flushed multiple times, large amounts of potentially infectious viral aerosols accumulate in public toilets, increasing the risk of airborne transmission. Kang et al. [46] found that viral aerosols were produced when the toilet was flushed, and aerosols of sufficiently small size are likely to be airborne in drainage pipes and vents for hours. The buoyancy effect may occur when the air temperature and humidity in the toilet change, and the use of exhaust fans may generate negative pressure, leading to aerosol diffusion to other floors of the same building. Boles et al. [40] used a Coriolis µ aerosol sampler to collect MNV-positive air samples around the toilet after flushing, with a viral concentration of 383–684 genome copy numbers/m^3^. The highest average particle concentration was observed behind the toilet and 0.15 m above the edge of the toilet. This study also showed that most aerosol particles produced by toilet flushing were smaller than 1 μm in size, with 0.3 μm particles being the most common. In addition, the use of squat toilets remains high worldwide, and we suspect that the concentration of NoV aerosols in bathrooms using squat toilets may be higher than in bathrooms using standard toilets; however, further research is needed to confirm this.

### 3.3. Cases of NoV Aerosol Transmission

Relatively few cases of NoV aerosol transmission have been reported compared to other transmission modes. As shown in Table 1, we collected typical aerosol transmission cases and summarized their location, number of cases, setting, time, source of infection, mode of transmission and evidence of aerosol transmission.

## 4. Norovirus Aerosol Collection

### 4.1. Common Collection Methods of Viral Aerosol

Currently, filtration retention and inertial impact methods are commonly used to collect viral aerosols [22]. According to the collection principle, the inertial impact method is divided into jet impact sampler and centrifugal cyclone sampler. According to the collection medium, the jet impact sampler can be further divided into solid impact sampler and liquid impact sampler. The collection medium of a solid impact sampler is usually a nutrient medium, which is more convenient for the collection and identification of bacteria, fungi and other microorganisms, and the collection efficiency of viruses is low, and the operation is complicated. Therefore, the collection of viral aerosols is usually performed using the filter, liquid impact or cyclone samplers. For example, Brooks and Bonifait et al. [30,31] successfully collected NoV aerosols in the air at biosolid land application sites and medical facilities using liquid impact and cyclone samplers, respectively. In the study of Masclaux, Tseng and Uhrbrand et al. [32,64,65,66], gelatin and teflon filters had also been successfully used to collect NoV and other enteric viruses in the air.

### 4.2. The Commonly Used NoV Aerosol Sampler

The Biosampler (SKC, Eighty Four, PA, USA) is one of the most commonly used liquid impact samplers for collecting bioaerosols, and it has been widely used to study a variety of microorganisms, including fungi, bacteria and viruses [67,68,69]. Although the SKC Biosampler microbial aerosol sampler can capture NoV, research showed that the sampler had a low collection efficiency for submicrometric and ultrafine particles, which often contain viruses, with a collection efficiency of less than 10% in the 30–100 nm size range [70]. This indicates that the sampler may underestimate the amount of viral aerosol. In addition, another disadvantage of liquid impact samplers is the loss of liquid medium due to evaporation. If the sampling time is too long and the liquid medium evaporates too much, it can affect the active state of the virus, and the captured virions may be aerosolized again [71,72].

Uhrbrand et al. [33] used four types of personal samplers (GSP sampler, TC three-stage cyclone sampler, 3P closed-face cassettes filter sampler and NIOSH cyclone sampler) combined with four filters (nylon, polycarbonate, teflon and gelatin) to collect aerosols. They found that the types of samplers and filters had a significant effect on the sampling efficiency for MNV aerosol. The combination of a GSP sampler and nylon filter had the highest collection efficiency for MNV aerosol; subsequently, the NoV aerosols were successfully collected in a hospital sewage treatment plant using this combination [32].

In addition, Matsubara et al. [35] developed a portable collection method (HA vortex method) for various enteric viruses (including NoV) in the air, and used a mixed cellulose membrane (HA 0.45 µm, Millipore, Billerica, MA, USA) as the collection medium and eluted the viruses using a glycine buffer. Its collection capability was similar to the liquid medium collection method (Biosampler, SKC) and had been successfully applied to detect enteric viruses at a sewage treatment plant in Japan. The method is simple and convenient, without on-site operation of personnel, and is suitable for sampling in sewage treatment plants where viruses are abundant in the environment. However, this method has shown false-negative results and therefore needs to be further optimized.

Boles et al. [73] found that a higher concentration of MNV was detected using a balanced salt solution (HBSS) as the medium when using a Biosampler liquid impaction sampler or NIOSH-251 cyclone sampler. However, the proportion of active MNV virions was higher when using a phosphate solution (PBS) as the medium. These results suggest that the appropriate sampling method and medium should be selected according to the purpose of the study.

## 5. Conclusions

NoV aerosol transmission often occurs simultaneously with person-to-person transmission, including the contact with infected individuals and contaminated environments. Therefore, a rigorous investigation and analysis are required when determining the transmission mode. There is also a lack of standard sampling equipment and procedures for viral aerosols, which hinders a better understanding of the source, concentration, particle size, residence time, movement and distribution of viral aerosols. In addition, we need to establish an effective in vitro culture system to evaluate the infectivity of NoV aerosols, which is another challenge that that is currently exists. 

At present, most cases of NoV aerosols are related to vomiting, and diarrhea-related ones are less common. Further research is needed to investigate the role of diarrhea and toilet flushing in the transmission of NoV aerosols, including the concentration of aerosols in toilets and their infectivity. Recent reports have indicated salivary transmission as a new mode of transmission [74]. Therefore, it is essential to explore whether activities such as coughing, talking, sneezing and other behaviors of infected individuals contribute to the aerosol transmission of NoV. It is necessary to further explore the aerosol transmission mechanism of NoV to provide a theoretical basis for formulating prevention and control policies.

## Figures and Tables

**Table 1:** NoV aerosol transmission cases.

Location	Number of Cases	Setting	Time	Infection Source	Mode of Transmission	Aerosol Transmission Evidence	References
Philadelphia, Pennsylvania, USA	NA	Medical College	1945	The patient’s gargle and stool suspension	airborne, fecal–oral	Of the 32 volunteers who inhaled gargles aerosol, 17 developed symptoms.	[47]
Toronto, Ontario, Canada	635	Hospital	01/11/1985–22/11/1985	NA	airborne	1. Among those who visited the emergency department from November 11 to 12, there was no correlation between the incidence and indirect contact behavior or diet, but there was a correlation with the length of stay in the emergency department. 2. The risk of illness for housekeepers who visited the emergency room was four times greater than those who did not.	[48]
Los Angeles, USA	NA	Geriatric convalescent institution	12/1988–01/1989	Infection may originate from hospitals and may be transmitted to the convalescent facility via cross-staff	multiple routes of transmission, including airborne transmission	1. Fourteen cases had no contact with patients or their stool. 2. Foodborne and waterborne transmission were ruled out.	[49]
UK	126	Nursing home	14/11/1992–03/12/1992	The virus was likely introduced from the community by staff or patients	airborne (aerosol-vomitus); environmental contamination	1. Exposure to nearby vomiting was the only risk factor. 2. No significant correlation was found between cleaning the patient’s vomit and feces and gastroenteritis among nurses.	[50]
UK	52	Hotel Restaurant	07/12/1998–11/12/1998	A guest who attended a party vomited at the restaurant	airborne (aerosol-vomitus)	1. None of the foods served at the dinner party were likely to be a vector of virus transmission. 2. The attendant who cleaned up the vomit did not fall ill. 3. There was a significant correlation between the distance between other guests and the vomiting location and their risk of illness. 4. No one at the restaurant became ill after the incident.	[24]
Derbyshire, England	153	Schools (including primary schools and nurseries for children aged 4 to 11)	25/06/2001–16/07/2001	NA	airborne (aerosol-vomitus); environmental contamination	1. Cleaned up immediately after vomiting. 2. Incidence increased with the number of exposures to vomiting. 3. In classrooms where three vomiting events occurred on the same day, the median time from exposure to onset was significantly shorter than those in the other two classrooms where vomiting occurred only once.	[25]
Japan	444	Hotel	02/12/2006–10/12/2006	The index case vomited in corridors on the 3rd and 25th floors	airborne (aerosol-vomitus); contact with fomites	1. NoV was detected in the contaminated carpet and also in the environment samples that were unlikely to be touched, such as mantels or light fittings. 2. Eighteen guests did not eat any of the party food, were still sick and were infected with the same NoV genotype as those who ate the party food. 3. Foodborne transmission was ruled out.	[51]
Salzburg Province, Austria	176	Youth hostel (holiday for teachers and students of 4 schools)	08/12/2007–17/12/2007	The first case vomited several times on a bus from Vienna to Salzburg Province on 8 December 2007, and vomited again in the hotel lobby after arrival	airborne (aerosol-vomitus); subsequent person-to-person transmission or exposure to a contaminated environment	1. A day-by-day analysis of specific foods showed that no food was associated with the risk of infection between 8 and 12 December. 2. Other transmission modes were ruled out.	[52]
Changzhou City, China	207	Primary school	22/11/2011–02/12/2011	The earliest cases vomited repeatedly in public places such as classrooms, bathrooms and staircases	airborne (aerosol-vomitus); person-to-person transmission or exposure to a contaminated environment	1. Exposure to vomiting and contact with patients were associated with an increased risk of NoV gastroenteritis. 2. Foodborne and waterborne transmission were ruled out. 3. Within the first 48 h, students closer to the site of vomiting fell ill earlier than those farther away.	[53]
Quebec City, Canada	NA	Eight medical facilities	2012	NA	airborne, other routes of transmission cannot be ruled out	1. NoV GII genomes were detected in 23 air samples from six healthcare facilities, with concentrations ranging from 1.35 × 10^1^ to 2.35 × 10^3^ genomes/m^3^. 2. In vitro experiments showed that MNV remained intact and infectious in aerosols.	[30]
Gothenburg, Sweden	NA	Sahel Greska University Hospital	2012	NA	multiple routes of transmission, including airborne transmission	The gene sequences of NoV GII.4 strains detected in dust, virus trap sampling device and air vents in the ward were the same or highly similar to those from the patients.	[54]
Yiyang City, Hunan Province, China	105	Senior high school	18/02/2014–02/03/2014	NA	airborne (aerosol-vomitus); contact with fomites	1. The student vomited on the floor of the classroom or the bathroom sink, and did not disinfect and clean up in time. The student washed the mop in the bathroom sink and moped the classroom floor. 2. Classrooms were grouped according to their distance from the bathroom; the farther the classroom was from the bathroom, the lower the incidence of illness.	[55]
Ningbo City, Zhejiang Province, China	46	Primary school	03/01/2015–13/01/2015	The first case may were infected by consuming oysters contaminated with NoV	airborne (aerosol-vomitus); person-to-person transmission	1. The vomit of the first case was not treated in a timely and standardized manner, and the vomit was only removed without disinfection. 2. Due to the rainy and cold weather, the classroom was not ventilated by opening windows.	[56]
Zhongshan City, Guangdong Province, China	39	Primary school	23/02/2016–29/02/2016	The indicative case vomited in the school hallway	airborne (aerosol-vomitus); person-to-person transmission	1. Passing through the vomit site was a risk factor. 2. The low wind in the vomit-contaminated area was not conducive to the rapid dispersal of aerosols.	[57]
Shanghai City, China	19	Primary school	07/03/2017–10/03/2017	The first case vomited at the playground	airborne (aerosol-vomitus)	1. The epidemic curve was unimodal, indicating the occurrence of point source exposure. 2. The first case vomited in the playground during physical education class, and the vomit was not disinfected in time.	[58]
Lianyungang, China	20	Kindergarten	09/06/2017–29/06/2017	The first case vomited on the top bunk of a bunk bed in the lunch break room	airborne (aerosol-vomitus)	1. The beds of all cases were located in the fan-shaped area covered by air conditioning exhaust. 2. Teachers and children who were not in the lunch break room on that day were not infected with NoV. 3. The outbreak occurred in only one class, and investigations showed lunch and drinking water were not risk factors.	[59]
Sweden	26	Hospitals (13 wards in 3 hospitals)	03/2017–05/2018	NA	airborne transmission; other transmission routes were not ruled out	1. Detection of NoV RNA in the air was associated with a shorter time from vomiting. 2. The concentration of NoV RNA in the air ranged from 5 to 215 copies/m^3^, with an average of 31 copies/m^3^, and NoV RNA was detected in aerosols with particle sizes of <0.95 µm and >4.51 µm.	[13]
Shenzhen City, Guangdong Province, China	16	Kindergarten	10/09/2018–12/09/2018	The first case vomited in the classroom during the nap time of young children	airborne (aerosol-vomitus)	1. The outbreak was characterized by point-source exposure. 2. During naptime, windows and doors were closed, and air conditioners were turned on; therefore, children and staff were in an enclosed space, and most cases were in the fan-shaped area covered by air conditioning exhaust. 3. The cleaning of vomit was not standardized.	[60]
Wenzhou City, Zhejiang Province, China	28	Junior high school	14/12/2018–24/12/2018	The first case vomited in the classroom	airborne (aerosol-vomitus); person-to-person transmission	1. The vomit from the first case was simply swept up and was not disinfected, and the cleaning tools were also not disinfected. 2. Cases were mainly concentrated around the first case and the vomit. 3. Cleaning up vomit and staying less than 2 m away from vomit are risk factors.	[61]
Wuhan City, Hubei Province, China	17	Kindergarten	16/11/2020–18/11/2020	The first case vomited in the classroom	airborne (aerosol-vomitus); person-to-person transmission	1. The vomit was not disposed of properly, and the disinfection concentration was not enough. 2. Students did not leave the classroom and were not ventilated before cleaning up the vomit. 3. The closer the student was to the vomit of the first case, the greater the risk of infection.	[62]
Xi’an City, Shaanxi Province, China	31	Kindergarten	26/05/2021–28/05/2021	The first case vomited in the classroom	airborne (aerosol-vomitus)	1. The risk of illness in children close exposed to vomit was 3.98 times higher than in children exposed at a distance, and 102 times higher than in unexposed children. 2. Vomit cleanup was not standardized, and the garbage bag containing the vomit was not sealed. 3. Contaminated areas were not disinfected, children were not immediately evacuated and cases were not isolated.	[63]

## References

[1] Pires S.M., Fischer-Walker C.L., Lanata C.F., Devleesschauwer B., Hall A.J., Kirk M.D., Duarte A.S., Black R.E., Angulo F.J. (2015). Aetiology-Specific Estimates of the Global and Regional Incidence and Mortality of Diarrhoeal Diseases Commonly Transmitted through Food. PLoS ONE.

[2] Lopman B.A., Steele D., Kirkwood C.D., Parashar U.D. (2016). The Vast and Varied Global Burden of Norovirus: Prospects for Prevention and Control. PLoS Med..

[3] Lysén M., Thorhagen M., Brytting M., Hjertqvist M., Andersson Y., Hedlund K.O. (2009). Genetic diversity among food-borne and waterborne norovirus strains causing outbreaks in Sweden. J. Clin. Microbiol..

[4] Wikswo M.E., Hall A.J. (2012). Outbreaks of acute gastroenteritis transmitted by person-to-person contact—United States, 2009–2010. MMWR Surveill Summ..

[5] Verhoef L., Hewitt J., Barclay L., Ahmed S.M., Lake R., Hall A.J., Lopman B., Kroneman A., Vennema H., Vinjé J. (2015). Norovirus genotype profiles associated with foodborne transmission, 1999–2012. Emerg. Infect. Dis..

[6] Atmar R.L., Opekun A.R., Gilger M.A., Estes M.K., Crawford S.E., Neill F.H., Graham D.Y. (2008). Norwalk virus shedding after experimental human infection. Emerg. Infect. Dis..

[7] Teunis P.F., Moe C.L., Liu P., Miller S.E., Lindesmith L., Baric R.S., Le Pendu J., Calderon R.L. (2008). Norwalk virus: How infectious is it?. J. Med. Virol..

[8] La Rosa G., Fratini M., Della Libera S., Iaconelli M., Muscillo M. (2013). Viral infections acquired indoors through airborne, droplet or contact transmission. Ann. Ist. Super. Sanita.

[9] Harris J.P., Lopman B.A., O’Brien S.J. (2010). Infection control measures for norovirus: A systematic review of outbreaks in semi-enclosed settings. J. Hosp. Infect..

[10] Gonzaga V.E., Ramos M., Maves R.C., Freeman R., Montgomery J.M. (2011). Concurrent outbreak of norovirus genotype I and enterotoxigenic Escherichia coli on a U.S. Navy ship following a visit to Lima, Peru. PLoS ONE.

[11] Hinds W.C. (1999). Aerosol Technology: Properties, Behavior, and Measurement of Airborne Particles.

[12] Jones R.M., Brosseau L.M. (2015). Aerosol transmission of infectious disease. J. Occup. Environ. Med..

[13] Alsved M., Fraenkel C.J., Bohgard M., Widell A., Soderlund-Strand A., Lanbeck P., Holmdahl T., Isaxon C., Gudmundsson A., Medstrand P. (2020). Sources of Airborne Norovirus in Hospital Outbreaks. Clin. Infect. Dis..

[14] Despres V.R., Huffman J.A., Burrows S.M., Hoose C., Safatov A.S., Buryak G., Froehlich-Nowoisky J., Elbert W., Andreae M.O., Poeschl U. (2012). Primary biological aerosol particles in the atmosphere: A review. Tellus Ser. B-Chem. Phys. Meteorol..

[15] Infection Prevention and Control of Epidemic-and Pandemic Prone Acute Respiratory Infections in Health Care. https://www.who.int/publications/i/item/infection-prevention-and-control-of-epidemic-and-pandemic-prone-acute-respiratory-infections-in-health-care.

[16] Gralton J., Tovey E., McLaws M.L., Rawlinson W.D. (2011). The role of particle size in aerosolised pathogen transmission: A review. J. Infect..

[17] Lipworth B., Manoharan A., Anderson W. (2014). Unlocking the quiet zone: The small airway asthma phenotype. Lancet Respir. Med..

[18] Atmar R.L., Opekun A.R., Gilger M.A., Estes M.K., Crawford S.E., Neill F.H., Ramani S., Hill H., Ferreira J., Graham D.Y. (2014). Determination of the 50% human infectious dose for Norwalk virus. J. Infect. Dis..

[19] Magill-Collins A., Gaither M., Gerba C.P., Kitajima M., Iker B.C., Stoehr J.D. (2015). Norovirus Outbreaks Among Colorado River Rafters in the Grand Canyon, Summer 2012. Wilderness Environ. Med..

[20] Kirby A.E., Streby A., Moe C.L. (2016). Vomiting as a Symptom and Transmission Risk in Norovirus Illness: Evidence from Human Challenge Studies. PLoS ONE.

[21] Morawska L. (2006). Droplet fate in indoor environments, or can we prevent the spread of infection?. Indoor Air.

[22] Verreault D., Moineau S., Duchaine C. (2008). Methods for sampling of airborne viruses. Microbiol. Mol. Biol. Rev..

[23] Ciofi-Silva C.L., Bruna C.Q.M., Carmona R.C.C., Almeida A., Santos F.C.P., Inada N.M., Bagnato V.S., Graziano K.U. (2019). Norovirus recovery from floors and air after various decontamination protocols. J. Hosp. Infect..

[24] Marks P.J., Vipond I.B., Carlisle D., Deakin D., Fey R.E., Caul E.O. (2000). Evidence for airborne transmission of Norwalk-like virus (NLV) in a hotel restaurant. Epidemiol. Infect..

[25] Marks P.J., Vipond I.B., Regan F.M., Wedgwood K., Fey R.E., Caul E.O. (2003). A school outbreak of Norwalk-like virus: Evidence for airborne transmission. Epidemiol. Infect..

[26] Isakbaeva E.T., Widdowson M.A., Beard R.S., Bulens S.N., Mullins J., Monroe S.S., Bresee J., Sassano P., Cramer E.H., Glass R.I. (2005). Norovirus transmission on cruise ship. Emerg. Infect. Dis..

[27] Gallimore C.I., Taylor C., Gennery A.R., Cant A.J., Galloway A., Xerry J., Adigwe J., Gray J.J. (2008). Contamination of the hospital environment with gastroenteric viruses: Comparison of two pediatric wards over a winter season. J. Clin. Microbiol..

[28] Tung-Thompson G., Libera D.A., Koch K.L., de Los Reyes F.L., Jaykus L.A. (2015). Aerosolization of a Human Norovirus Surrogate, Bacteriophage MS2, during Simulated Vomiting. PLoS ONE.

[29] Kramer A., Schwebke I., Kampf G. (2006). How long do nosocomial pathogens persist on inanimate surfaces? A systematic review. BMC Infect. Dis..

[30] Bonifait L., Charlebois R., Vimont A., Turgeon N., Veillette M., Longtin Y., Jean J., Duchaine C. (2015). Detection and quantification of airborne norovirus during outbreaks in healthcare facilities. Clin. Infect. Dis..

[31] Brooks J.P., Tanner B.D., Josephson K.L., Gerba C.P., Haas C.N., Pepper I.L. (2005). A national study on the residential impact of biological aerosols from the land application of biosolids. J. Appl. Microbiol..

[32] Uhrbrand K., Schultz A.C., Koivisto A.J., Nielsen U., Madsen A.M. (2017). Assessment of airborne bacteria and noroviruses in air emission from a new highly-advanced hospital wastewater treatment plant. Water Res..

[33] Uhrbrand K., Koponen I.K., Schultz A.C., Madsen A.M. (2018). Evaluation of air samplers and filter materials for collection and recovery of airborne norovirus. J. Appl. Microbiol..

[34] Alsved M., Widell A., Dahlin H., Karlson S., Medstrand P., Londahl J. (2020). Aerosolization and recovery of viable murine norovirus in an experimental setup. Sci. Rep..

[35] Matsubara K., Katayama H. (2019). Development of a Portable Detection Method for Enteric Viruses from Ambient Air and Its Application to a Wastewater Treatment Plant. Pathogens.

[36] Suffredini E., Pepe T., Ventrone I., Croci L. (2011). Norovirus detection in shellfish using two Real-Time RT-PCR methods. New Microbiol..

[37] Taube S., Kolawole A.O., Höhne M., Wilkinson J.E., Handley S.A., Perry J.W., Thackray L.B., Akkina R., Wobus C.E. (2013). A mouse model for human norovirus. mBio.

[38] Cromeans T., Park G.W., Costantini V., Lee D., Wang Q., Farkas T., Lee A., Vinjé J. (2014). Comprehensive comparison of cultivable norovirus surrogates in response to different inactivation and disinfection treatments. Appl. Environ. Microbiol..

[39] Manuel C.S., Moore M.D., Jaykus L.A. (2015). Destruction of the Capsid and Genome of GII.4 Human Norovirus Occurs during Exposure to Metal Alloys Containing Copper. Appl. Environ. Microbiol..

[40] Boles C., Brown G., Nonnenmann M. (2021). Determination of murine norovirus aerosol concentration during toilet flushing. Sci. Rep..

[41] Johnson D.L., Mead K.R., Lynch R.A., Hirst D.V. (2013). Lifting the lid on toilet plume aerosol: A literature review with suggestions for future research. Am. J. Infect. Control.

[42] Barker J., Jones M.V. (2005). The potential spread of infection caused by aerosol contamination of surfaces after flushing a domestic toilet. J. Appl. Microbiol..

[43] Knowlton S.D., Boles C.L., Perencevich E.N., Diekema D.J., Nonnenmann M.W. (2018). Bioaerosol concentrations generated from toilet flushing in a hospital-based patient care setting. Antimicrob. Resist. Infect. Control.

[44] Verani M., Bigazzi R., Carducci A. (2014). Viral contamination of aerosol and surfaces through toilet use in health care and other settings. Am. J. Infect. Control.

[45] Schreck J.H., Lashaki M.J., Hashemi J., Dhanak M., Verma S. (2021). Aerosol generation in public restrooms. Phys. Fluids.

[46] Kang M., Wei J., Yuan J., Guo J., Zhang Y., Hang J., Qu Y., Qian H., Zhuang Y., Chen X. (2020). Probable Evidence of Fecal Aerosol Transmission of SARS-CoV-2 in a High-Rise Building. Ann. Intern. Med..

[47] Reimann H.A., Price A.H., Hodges J.H. (1945). The Cause of Epidemic Diarrhea, Nausea and Vomiting. (Viral Dysentery?). Exp. Biol. Med..

[48] Sawyer L.A., Murphy J.J., Kaplan J.E., Pinsky P.F., Chacon D., Walmsley S., Schonberger L.B., Phillips A., Forward K., Goldman C. (1988). 25- to 30-nm virus particle associated with a hospital outbreak of acute gastroenteritis with evidence for airborne transmission. Am. J. Epidemiol..

[49] Gellert G.A., Waterman S.H., Ewert D., Oshiro L., Giles M.P., Monroe S.S., Gorelkin L., Glass R.I. (1990). An outbreak of acute gastroenteritis caused by a small round structured virus in a geriatric convalescent facility. Infect. Control Hosp. Epidemiol..

[50] Chadwick P.R., McCann R. (1994). Transmission of a small round structured virus by vomiting during a hospital outbreak of gastroenteritis. J. Hosp. Infect..

[51] Kimura H., Nagano K., Kimura N., Shimizu M., Ueno Y., Morikane K., Okabe N. (2011). A norovirus outbreak associated with environmental contamination at a hotel. Epidemiol. Infect..

[52] Kuo H.W., Schmid D., Schwarz K., Pichler A.M., Klein H., Konig C., de Martin A., Allerberger F. (2009). A non-foodborne norovirus outbreak among school children during a skiing holiday, Austria, 2007. Wien. Klin. Wochenschr..

[53] Xu H., Lin Q., Chen C., Zhang J., Zhang H., Hao C. (2013). Epidemiology of norovirus gastroenteritis outbreaks in two primary schools in a city in eastern China. Am. J. Infect. Control.

[54] Nenonen N.P., Hannoun C., Svensson L., Torén K., Andersson L.M., Westin J., Bergström T. (2014). Norovirus GII.4 detection in environmental samples from patient rooms during nosocomial outbreaks. J. Clin. Microbiol..

[55] Hu H.A., Zhang Y.B., Luo J.H., Jiang Z.H., Lu Y., Gong Q., Chen Y., Hu S.X., Luo K.W. (2015). An outbreak of infectious diarrhea with noroviruses caused by poor toilet hygiene. Pract. Prev. Med..

[56] Liu S.K., Zhang H.B., Zhang F., Wang F., Hu L.L., You L.X. (2016). Investigation of a school outbreak of norovirus diarrhea. Prev. Med..

[57] Luo L., Wang M., Zhu Y.Y., Lin Y.Y. (2017). Investigation of an outbreak caused by transmission of norovirus in Zhongshan city. J. Trop. Med..

[58] Mo Y.J., Wang X.L., Yan R.Z. (2018). Analysis of epidemiological characteristics of a cluster of infectious diarrhea caused by norovirus type GII in a school in Jiading District, Shanghai. Shanghai Med. Pharm. J..

[59] Zhang T.L., Lu J., Ying L., Zhu X.L., Zhao L.H., Zhou M.Y., Wang J.L., Chen G.C., Xu L. (2017). An acute gastroenteritis outbreak caused by GII.P16-GII.2 norovirus associated with airborne transmission via the air conditioning unit in a kindergarten in Lianyungang, China. Int. J. Infect. Dis..

[60] Wang K.L., Huang Z.H., Pan L.L., Jin H.L., Luo R.Y., Chen H.B. (2019). Investigation and analysis of a clustering event of infectious diarrhea by Norovirus type Ⅱ in a kindergarten. Anhui J. Prev. Med..

[61] Ni C.R., Wang J., Pan Q.J., Xu F., Xiang F.L., Ying S.Y. (2019). Outbreak investigation of acute gastroenteritis associated with norovirus in Wenzhou. Chin. J. Public Health Manag..

[62] Wu L., Tian W.D., Li H., Dong M.N. (2021). Investigation and analysis of a cluster epidemic of norovirus infection in kindergarten. Anhui J. Prev. Med..

[63] Zhang H., Li Q., Tang H., Liu J.F., Li S.Y., Li Y. (2022). Investigation of a norovirus infection outbreak in a kindergarten and analysis on pathogen molecular epidemiological characteristics. Dis. Surveill..

[64] Masclaux F.G., Hotz P., Gashi D., Savova-Bianchi D., Oppliger A. (2014). Assessment of airborne virus contamination in wastewater treatment plants. Environ. Res..

[65] Tseng C.C., Chang L.Y., Li C.S. (2010). Detection of airborne viruses in a pediatrics department measured using real-time qPCR coupled to an air-sampling filter method. J. Environ. Health.

[66] Uhrbrand K., Schultz A.C., Madsen A.M. (2011). Exposure to Airborne Noroviruses and Other Bioaerosol Components at a Wastewater Treatment Plant in Denmark. Food Environ. Virol..

[67] Farnsworth J.E., Goyal S.M., Kim S.W., Kuehn T.H., Raynor P.C., Ramakrishnan M.A., Anantharaman S., Tang W. (2006). Development of a method for bacteria and virus recovery from heating, ventilation, and air conditioning (HVAC) filters. J. Environ. Monit..

[68] Hermann J.R., Hoff S.J., Yoon K.J., Burkhardt A.C., Evans R.B., Zimmerman J.J. (2006). Optimization of a sampling system for recovery and detection of airborne porcine reproductive and respiratory syndrome virus and swine influenza virus. Appl. Environ. Microbiol..

[69] Lindsley W.G., Blachere F.M., Thewlis R.E., Vishnu A., Davis K.A., Cao G., Palmer J.E., Clark K.E., Fisher M.A., Khakoo R. (2010). Measurements of airborne influenza virus in aerosol particles from human coughs. PLoS ONE.

[70] Hogan C.J., Kettleson E.M., Lee M.H., Ramaswami B., Angenent L.T., Biswas P. (2005). Sampling methodologies and dosage assessment techniques for submicrometre and ultrafine virus aerosol particles. J. Appl. Microbiol..

[71] Stewart S.L., Grinshpun S.A., Willeke K., Terzieva S., Ulevicius V., Donnelly J. (1995). Effect of impact stress on microbial recovery on an agar surface. Appl. Environ. Microbiol..

[72] Wang Z., Reponen T., Grinshpun S., Górny R., Willeke K. (2001). Effect of sampling time and air humidity on the bioefficiency of filter samplers for bioaerosol collection. J. Aerosol Sci..

[73] Boles C., Brown G., Park J.H., Nonnenmann M. (2020). The Optimization of Methods for the Collection of Aerosolized Murine Norovirus. Food Environ. Virol..

[74] Ghosh S., Kumar M., Santiana M., Mishra A., Zhang M., Labayo H., Chibly A.M., Nakamura H., Tanaka T., Henderson W. (2022). Enteric viruses replicate in salivary glands and infect through saliva. Nature.

